# Angelman Syndrome: A Case Report

**Published:** 2016

**Authors:** Farah Ashrafzadeh, Arianeh Sadrnabavi, Javad Akhondian, Mehran Beiraghi Toosi, Mohammadhassan Mohammadi, Kazem Hassanpour

**Affiliations:** 1 Department of Pediatric Neurology, Ghaem Medical Center, Mashhad University of Medical Sciences, Mashhad, Iran; 2 Dept. of Human Genetics, School of Medicine, Mashhad University of Medical Sciences, Mashhad, Iran; 3 Department of Pediatric, School of Medicine, Sabzevar University of Medical Sciences, Sabzevar, Iran

**Keywords:** Angelman syndrome, Child, Developmental delay, Iran

## Abstract

**Objective**

Angelman syndrome (AS) is a neurodevelopmental disorder presented by jerky movement, speech delay and cognitive disability epilepsy as well as dysmorphic features. It occurs due to an expression deletion in 15q11-q13 chromosome. In this article, we present an eight yr boy referred to Pediatrics Neurologic Clinic Mashhad, Iran for speech delay. He had abnormal behavior ataxia unusual laughing facial expression intellectual disability and mandibular prognathism.

Metabolic screening tests and brain MRI were normal. Genetic analysis was pathognomonic for AS.

## Introduction

Angelman syndrome (AS) was first described in 1965 by Harry Angelman (1).

He reported 3 children of healthy parents with neurologic syndrome of mental retardation, seizure, facial abnormalities, ataxia, speech disorders and unprovoked laugh pattern. He named them happy puppet. The name of this syndrome changed into AS in 1987 (2). Its genetic base was identified in 1987 (2).

AS is originated from dysfunction in expression of ubiquitin protein ligase E3A (UBE3A) gene in chromosome 15 (3). In 75% of cases a deletion happens in this chromosome, other changes such as translocation, mutation and micro-deletion are seen as well (4). These changes lead to malfunction of neurons. AS incidence is estimated 1 in 15000 live births, and recently nearly 450 cases of AS have been reported (5).

This neurodevelopmental syndrome occurs because of maternally inherited genes. 

Disease manifestations are language deficit, laughing facial expression, autistic or stereotyped behavior, jerky ataxia and severe mental retardation. Patients have communicative disability with or without seizure (3). These children also have cognitive disorders.

Here we describe a child with AS.

## Case report

An 8 yr old boy was referred to Pediatrics Neurologic Clinic Mashhad, Iran; due to developmental delay and seizure attacks from 6 yr ago. He was the second child of non relative healthy parents; there was no evidence of seizure or mental retardation in his family. He was born at term by a vaginal delivery with normal APGAR score and birth weight. He had a history of neonatal icterus and phototherapy.

He had a happy face and the parents noticed developmental delay at 2 yr old. He was admitted in a hospital because of tonic-colonic seizures and valproate sodium was administered for him. He was able to walk independently at 4 yr old. At the age of 6 yr, he was referred to Children Neurology Department in Mashhad due to hyperactivity, where received risperidone. 

**Fig 1:**
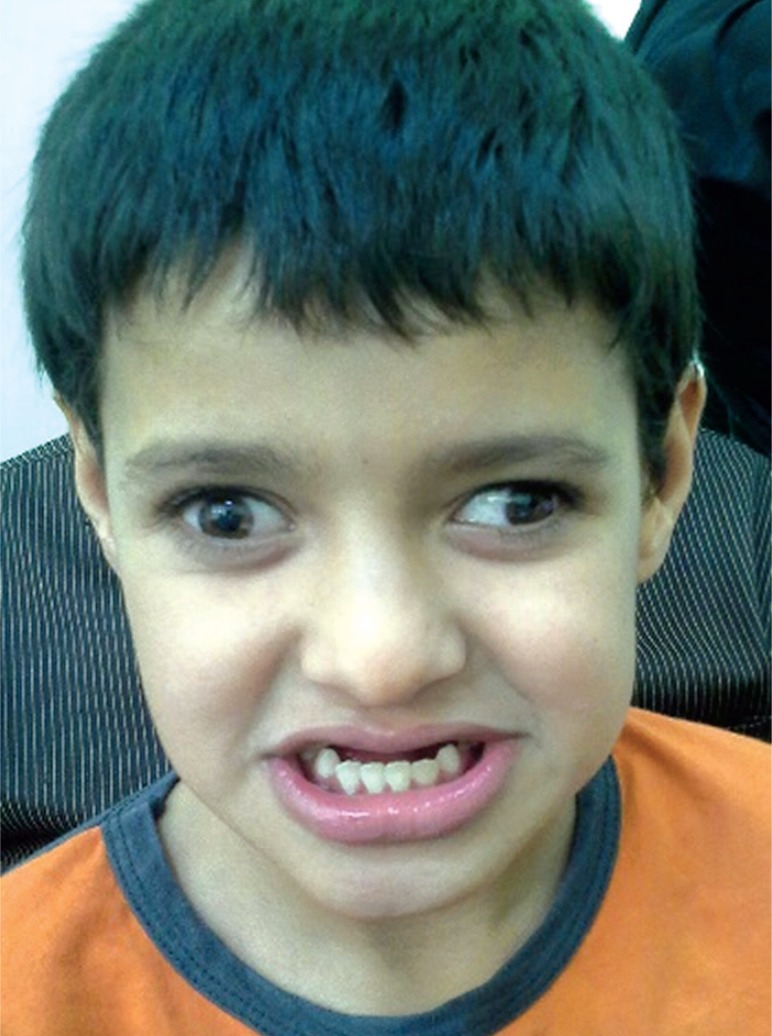
facial expression of Angelman syndrome

On physical examination, he had mandibular prognathism, strabismus and unusual laughing facial expression (Figure1). His head circumference was 51 cm.

Written informed consent was obtained from his parents.

His walking was unsteady, but muscles tone, force and deep tendon reflexes were normal. Joints range of motion was normal. Besides, he had speech disability and could walk independently, but could not run. He had restricted communicative abilities and suffered from severe mental retardation. 

Laboratory findings were normal. Brain MRI and CBC test were normal. Thyroid function test showed hypothyroidism, controlled by levothyroxine since 2 yr old.

In our patient, seizure attacks have been continued from the age of 2 yr, despite various pharmacologic treatments.

**Genetic findings**

After genomic DNA extraction, the DNAs were treated by bisulfide method and methylation specific PCR for the SRNP region were used. Genomic DNA was extracted from leukocytes collected in EDTA tubes (5PRIME kit GmbH, D-22767 Hamburg). The genomic DNA was treated by the Na bisulfit (So2) (Sigma) method. After treatment the methylation specific PCR was used.

The previously described primers, MAT: 5’-tat tgc ggt aaa taa gta cgt ttg cgc ggt c-3’ PAT: 5’-gtg agt ttg gtg tag agt gga gtg gtt gtt g-3’ COM: 5’-ctc caa aac aaa aaa ctt taa aac cca aat tcc-3’ were used to amplify the region.

Genomic treated DNA (300 ng) was used as template in a reaction volume of 23 μL, containing 10.8 μl ddH2O, 2.5 μl 10X reaction buffer, 25 mM MgCl2, and 10 mM dNTP, 5mM of each primer and 5U of Taq DNA polymerase (Genet Bio). Cycling conditions were 94 °C for 5 min, followed by 35 cycles of 25 sec at 94 °C, 25 sec at 60 °C and 25 sec at 72 °C, and 5 min at 72 °C as the final extension step. For the positive controlled of methylation test, we used the p16 gene.

**Fig 2:**
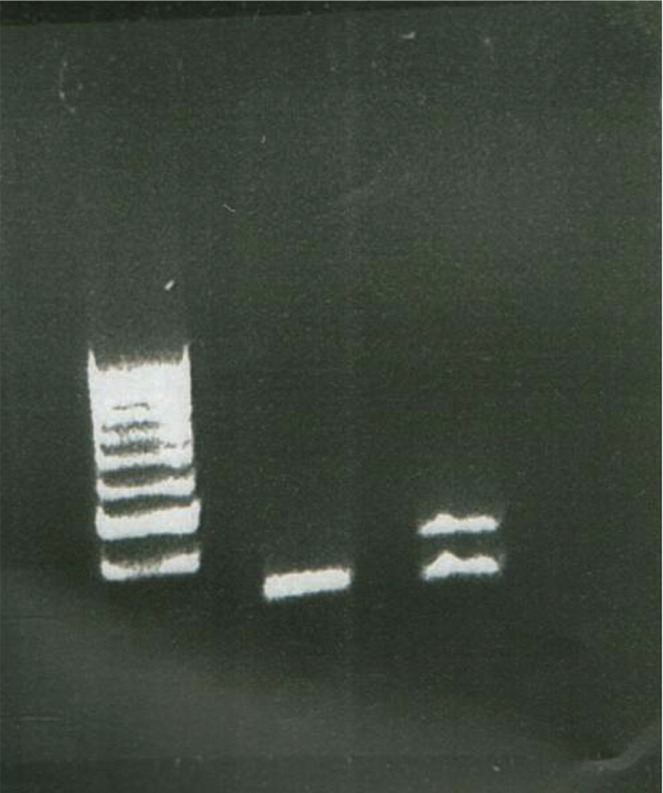
Agarose gel (2.5%) electrophoresis of BST PCR. 100 bp DNA ladder (Fermentas) was used as marker of DNA fragment sizes. Line 1. BST PCR from Engelmann Syndrome Patient. Line 2 BST PCR from Normal Person

For this patient abnormal methylation pattern of the SRNP region (Maternal imprinting defects or deletion) was detected. Therefore, an AS caused by micro deletion, uniparental disomie or imprinting defect (ID) was included. ID defect account for approximaly 3% of affected individual. ID have abnormal DNA methylation and 10-20% of the ID are caused by micro deletion (6-200kb) that include the imprinting Center (Figure 2).

## Discussion

Many cases of AS have impaired communication, which might occur due to language deficit and mental retardation (9). Our patient had this problem. 

Delayed motor development is common in AS, jerky movements are the first manifestation of AS, recognize by parents (10). In our reported case, developmental delayed was identified by parents at the age of 2 yr. 

Several facial abnormalities were reported in AS patients such as wide mouth, abnormal teeth, tongue protrusion and mandibular prognathism. There are some evidence on poor sucking and chewing in infancy (11). Our case had a wide mouth with unusual laughing lips, which is common in AS.

Around 70% of AS children could walk at 3 yr old (12), our caes could walk independently when he was 4 yr, but he still cannot run at 8.

Electroencephalography findings were abnormal in AS patient and EEG showed generalized spike and wave pattern (6). Over 855 of AS patients develop seizure in the first 3 yr of life. Its onset varies from 1 month to 20 years (7). Epilepsy is severe in AS cases and is hard to control as our case. It seems that valproic acid is the therapeutic choice in AS epilepsy (8).

Genetic tests for diagnosing AS are complex analysis. A small proportion of patients with AS may have a small deletion or other mutations that leads to aberrant imprinting of the region (13). These findings have led to major advances in genetic diagnosis of AS. 

Chromosome 15 deletions usually are submicroscopic but are easily detected by FISH and/or array CGH. 

Defects in imprinting or uniparental disomy can be identified by studies of patterns of DNA methylation in the region. Failure to identify the methylated or nonmethylated copy of the sequence is indicative of deletion, uniparental disomy, or a mutation that alters the imprinting mechanism. Genomic imprints are reversible and lead to differential expression in the course of development. Genomic imprinting is an epigenetic process that involves methylation and histone modifications in order to achieve monoallelic gene expression without altering the genetic sequence (14).

**In conclusion, **all physicians should consider rare syndromes such as AS in children or adults with neurodevelopmental delay. Noting clinical presentation is very important, because clinical suspicions play a crucial role to choose the required laboratory tests. On the other hand, multidiscipline approach is necessary for genetic syndromes like AS because they influence

all aspects of patients’ life.
